# Genome-Wide Analysis of the Gibberellin-Oxidases Family Members in Four *Prunus* Species and a Functional Analysis of *PmGA2ox8* in Plant Height

**DOI:** 10.3390/ijms25168697

**Published:** 2024-08-09

**Authors:** Xue Li, Jie Zhang, Xiaoyu Guo, Like Qiu, Ke Chen, Jia Wang, Tangren Cheng, Qixiang Zhang, Tangchun Zheng

**Affiliations:** Beijing Key Laboratory of Ornamental Plants Germplasm Innovation & Molecular Breeding, National Engineering Research Center for Floriculture, State Key Laboratory of Efficient Production of Forest Resources, Beijing Laboratory of Urban and Rural Ecological Environment, Engineering Research Center of Landscape Environment of Ministry of Education, Key Laboratory of Genetics and Breeding in Forest Trees and Ornamental Plants of Ministry of Education, School of Landscape Architecture, Beijing Forestry University, Beijing 100083, China; lx99092103@163.com (X.L.); zhangjie737373@126.com (J.Z.); karenkwok0517@163.com (X.G.); qiu_like@163.com (L.Q.); chenk200006@163.com (K.C.); wangjia@bjfu.edu.cn (J.W.); chengtangren@bjfu.edu.cn (T.C.); zqx@bjfu.edu.cn (Q.Z.)

**Keywords:** *Prunus mume*, gibberellin-oxidase, *PmGA2ox8*, gene expression, plant height

## Abstract

Gibberellins (GAs), enzymes that play a significant role in plant growth and development, and their levels in plants could be regulated by gibberellin-oxidases (GAoxs). As important fruit trees and ornamental plants, the study of the mechanism of plant architecture formation of the *Prunus* genus is crucial. Here, 85 *GAox* genes were identified from *P. mume*, *P. armeniaca*, *P. salicina*, and *P. persica*, and they were classified into six subgroups. Conserved motif and gene structure analysis showed that GAoxs were conserved in the four *Prunus* species. Collinearity analysis revealed two fragment replication events of *PmGAox*s in the *P. mume* genome. Promoter cis-elements analysis revealed 24 *PmGAox*s contained hormone-responsive elements and development regulatory elements. The expression profile indicated that *PmGAox*s have tissue expression specificity, and GA levels during the dormancy stage of flower buds were controlled by certain *PmGAox*s. After being treated with IAA or GA_3_, the transcription level of *PmGA2ox8* in stems was significantly increased and showed a differential expression level between upright and weeping stems. GUS activity driven by *PmGA2ox8* promoter was detected in roots, stems, leaves, and flower organs of *Arabidopsis*. *PmGA2ox8* overexpression in *Arabidopsis* leads to dwarfing phenotype, increased number of rosette leaves but decreased leaf area, and delayed flowering. Our results showed that GAoxs were conserved in *Prunus* species, and *PmGA2ox8* played an essential role in regulating plant height.

## 1. Introduction

Gibberellins (GAs), a kind of hormone widely existing in plants, plays an indispensable role in the stress response and growth of plants, such as seed germination, root and stem growth, and leaf and fruit development [1,2,3,4,5,6]. At present, a total of 136 types of GAs have been identified, which can be divided into C19-GAs groups and C20-GAs groups according to the number of carbon atoms [7,8]. However, only GA_1_, GA_3_, GA_4_, and GA_7_ are biologically active; the rest are intermediates in gibberellin synthesis or metabolism (Olszewski et al., 2002). The synthesis of bioactive and most intermediate GAs is catalyzed by GA 20-oxidases (GA20ox) and GA 3-oxidases (GA3ox), while GA 2-oxidases (GA2ox) catalyze GA deactivation [9,10]. These oxidases belong to the 2OG-Fe (II) oxygenase superfamily. The gene encoding GA2ox was initially obtained from runner beans, and *GA3ox* and *GA20ox* were isolated from *Arabidopsis* and pumpkin, respectively [11,12,13,14]. The whole genome identification of *GA2ox*, *GA3ox*, and *GA20ox* has been performed in some woody plants, such as grape, apple, tulip tree, and *Camellia sinensis* [15,16,17,18]. Moreover, functional analyses of *GAox*s have been performed in numerous woody plants [19,20,21,22].

GA20ox enzymes are mainly involved in the process of C20-GAs removing C atoms attached to C-20 to convert into C19-GAs. Two crucial bioactive GA precursors, GA_9_ and GA_20_, are produced by this process [23,24]. The well-known “Green Revolution” in rice breeding was due to the mutation of *OsGA20ox2*, which resulted in the *sd1* mutant exhibiting excellent traits such as short stature, thick stem, and lodging resistance [25,26]. *PdGA20ox1* from *Pinus densiflora* transformed *Arabidopsis* and *Populus*, resulting in accelerated stem growth and increased biomass of the two transgenic plants [20]. Inactive GA_9_ or GA_20_ can be converted to active GA_4_ or GA_1_ via a 3β-hydroxylation pathway catalyzed by GA3ox [23,24]. In *Arabidopsis*, *AtGA3ox3* and *AtGA3ox4* were involved in the production of GAs for reproductive development, while *AtGA3ox1* and *AtGA3ox2* function in the whole growth and development process of plants. *AtGA3ox1* and *AtGA3ox2* double mutants exhibited dwarfing phenotypes, and seed germination and root growth were impeded [27]. Similarly, pumpkins (*Cucurbita moschata*) exhibiting dwarfing phenotypes were found to be due to mutations in the *GA3ox* homologs [28]. Bioactive GA_4_ and GA_1_ can be transformed into inactive GAs by GA2ox. According to the different substrates, GA2ox can be divided into C19-GA2ox and C20-GA2ox, which act on the C19-GAs and C20-GAs precursor substances, respectively [24]. *GA2ox* in a transgenic dwarf poplar (*Populus tremula* × *Populus alba*) was considered to be a key gene that causes dwarfing [29]. Many studies have shown that overexpression of *GA2ox* in plants such as *Arabidopsis*, rice, tobacco, and soybean can lead to dwarfing phenotypes [30,31,32].

*Prunus mume*, the celebrated ornamental flower tree, originated in China and has a history of over 3000 years. *P. mume* can be divided into straight branch, weeping branch, and tortuous-branch types according to the tree structure. Numerous studies have shown that *GAox* genes have a significant impact on the formation of plant architecture, but there are currently no reports on the *GAox* family in *P. mume*. Here, we identified the members of *GAox* family in four *Prunus* species. Comprehensive analysis including physicochemical properties, gene structure, protein characteristics, and evolutionary relationships were explored. The cis-elements of promoter region and expression profiles of *PmGAox*s were also investigated. Furthermore, the coding sequence and promoter sequence of *PmGA2ox8* were cloned and transformed into plants, respectively. Overexpression of *PmGA2ox8* in *Arabidopsis* displayed a typical dwarf phenotype, which has great application value in *P. mume* and provides a great reference for developing a key candidate gene of plant architecture.

## 2. Results

### 2.1. Genome-Wide Identification and Analysis of GAoxs in Four Prunus Species

Eighty-five *GAox* genes were identified, with 24, 18, 22, and 21 in *P. mume*, *P. armeniaca*, *P. salicina,* and *P. persica*, respectively (Table S1). The encoding sequence for *GAox*s in four *Prunus* species ranged from 663 (*PmGA20ox3*) to 2163 bp (*PsGA2ox8*). Most GAox proteins had 220–434 amino acids (aa), and their molecular weight (MW) was 24.88 kDa–47.93 kDa, while PsGAox8 possessed the highest amino acids number (720 aa) and the largest MW (83.10 kDa). The predicted values of *pI* ranged from 4.80 to 8.79, and most proteins were acidic. Furthermore, subcellular localization predicted that all GAox proteins in four *Prunus* species were located in the cytoplasm. Chromosome mapping showed that most *GAox*s in the four species were distributed on chromosomes, while *PmGA2ox9*, *PaGA20ox6,* and *PsGA20ox8* were located on the scaffold.

**Figure 1:**
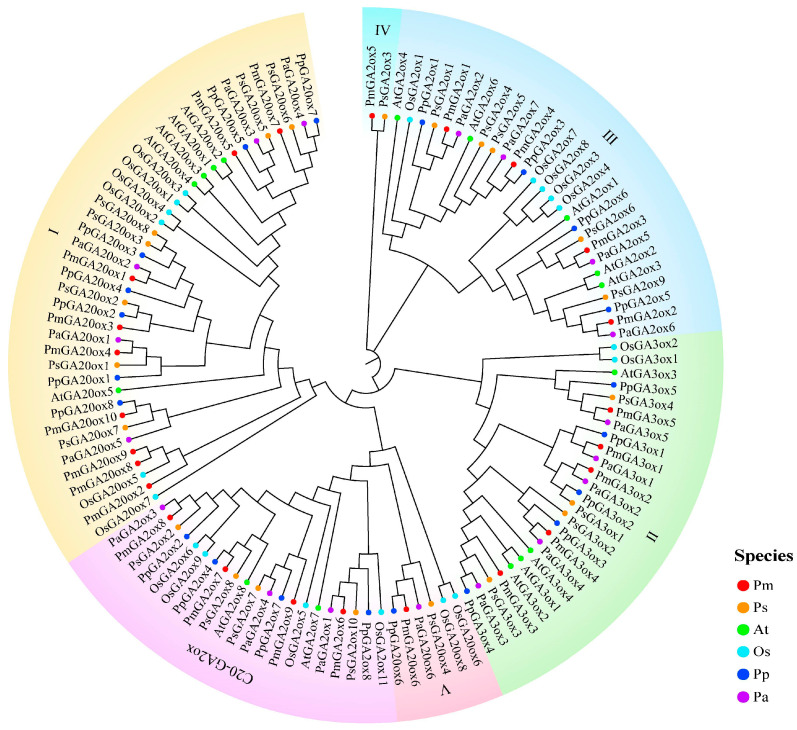
The NJ phylogenetic tree of GA2oxs in six species. At: *A. thaliana*; Os: *O. sativa*, Pm: *P. mume*, Pa: *P. armeniaca*, Ps: *P. salicina*, Pp: *P. persica*. The I, II, III, IV, V and C20-GA2ox represent group I, group II, group III, group IV, group V, and group C20-GA2ox, respectively. The phylogenetic tree was constructed based on GAox protein sequences from six species including Arabidopsis (16), rice (19), *P. mume* (24), *P. armeniaca* (18), *P. salicina* (22), and *P. persica* (21), using the NJ method in MEGA X with 1000 bootstrap replicates.

To investigate the evolutionary relationship of GAox in *Prunus* species, monocotyledons, and dicotyledons, the GAox protein sequences of *P. mume* (Pm), *P. armeniaca* (Pa), *P. salicina* (Ps), *P. persica* (Pp), rice (Os), and *Arabidopsis* (At) were used for phylogenetic analysis and construction of NJ tree (Figure 1). Based on the resulting NJ tree and the previous classifications of the GAox proteins in rice, 120 GAoxs of six species were divided into six subclasses, namely group I, group II, group III, group IV, group V, and group C20-GA2ox. Group I was the largest clade, containing most of the GA20ox proteins in six species. However, six GA20ox proteins (OsGA20ox6 and OsGA20ox8, PmGA20ox6, PaGA20ox6, PpGA20ox6, and PsGA20ox4) were classified as the group V, and they exhibit greater similarity to C20-GA2ox than to most GA20ox proteins. Group II and group C20-GA2ox contained all GA3ox and C20-GA2ox of these six species, respectively. Most C19-GA2ox were clustered in group III, but two proteins (PmGA2ox8 and PsGA2ox3) were separately clustered in Group IV. These two proteins had the longest branches, and no highly similar sequences were found in the model plant, possibly because some genes in *P. mume* and *P. salicina* originated relatively earlier and were not involved in evolution.

### 2.2. Gene Structure and Conserved Motif Analysis of GAox in Four Prunus Species

**Figure 2:**
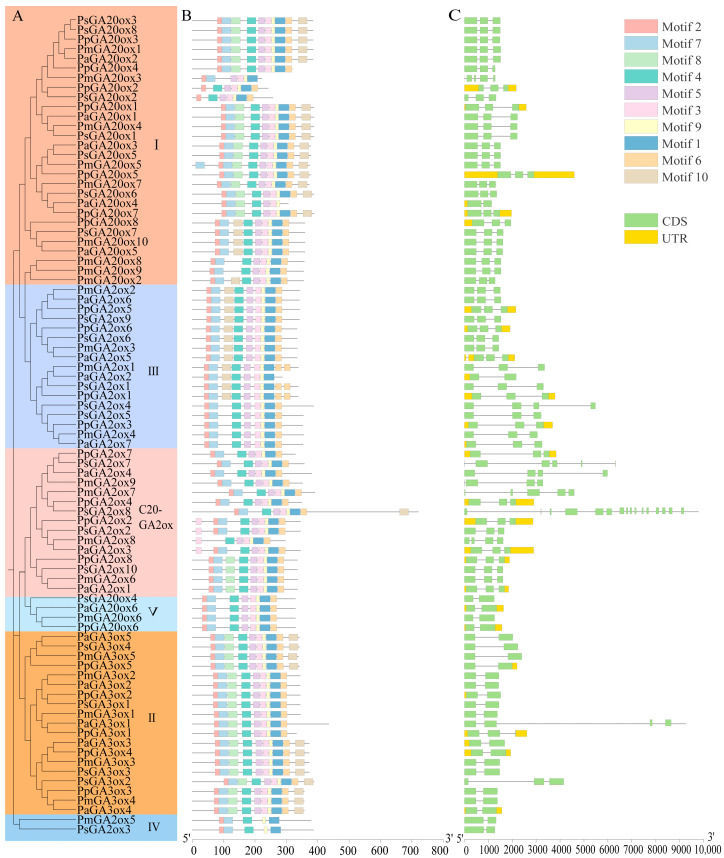
Evolutionary relationships, conserved motifs, and gene structure in GAox family members of four *Prunus* species. (**A**) The phylogenetic tree was constructed based on GAox protein sequences from four *Prunus* species. (**B**) The conserved motifs of GAox proteins in four *Prunus* species; different-colored boxes represent different motif. (**C**) Exon–intron structure of *GAox*s. Black lines indicate introns. The I, II, III, IV, V and C20-GA2ox represent group I, group II, group III, group IV, group V, and group C20-GA2ox, respectively.

GAox with closer evolutionary relationships had similar conserved motif distributions (Figure 2A,B). Motifs 1, 2, 3, 4, 5, 6, 7, and 9 were present in most members, indicating that the GAoxs were highly conserved and had a certain degree of functional similarity. Motif 1 and Motif 2 belonged to the 2OG-FeIIoxygenase and DIOX_N domains, respectively. These two domains were conserved in GAox family and played a crucial role in GA synthesis and metabolism. Some motifs exist in specific subfamilies. For example, Motif 8 was absent in group III, group IV and group V, while all members in group II possess it. C20-GA2ox lacks motif 10, except for PsGA2ox8 with an amino acid number exceeding 700 aa, which may be due to fragment loss events that have occurred during evolution. The coding sequence length and exons number of *GAox*s were diverse (Figure 2C). Most *GAox*s contained two to three exons, but some C20-GA2ox had more than four exons, such as *PsGA2ox7* (6), *PmGA2ox7* (5), and *PsGA2ox8* (16). More genes are less than 3 kb in length and have introns no longer than 2 kb, but some genes, most of which were *GA2ox*, have a length of over 3 kb and longer introns.

### 2.3. Synteny Analysis of GAoxs in Four Prunus Species

**Figure 3:**
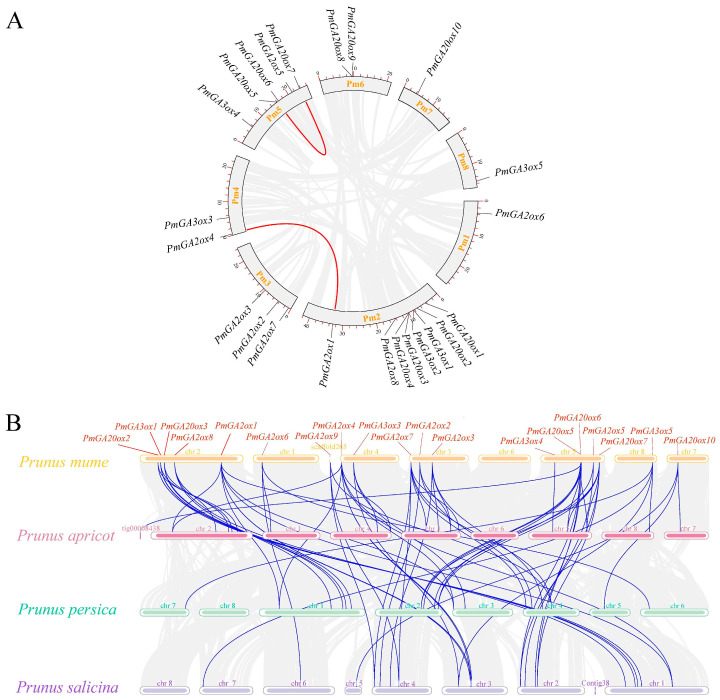
Collinear analysis of *GAox* genes. (**A**) Distribution segmental duplication of *GAox* genes in *P. mume*. Gray lines indicate all collinear blocks within *P. mume*, and the red lines indicate segmental duplicated *PmGAox* gene pairs. (**B**) Synteny analysis of *GAox* genes between *P. mume* and three *Prunus* species (*P. armeniaca*, *P. salicina*, and *P. persica*). Gray lines in the background indicate the collinear blocks throughout the genome, and blue lines represent the syntenic *GAox* gene pairs.

The syntenic regions within the *P. mume* genome were analyzed to reveal the duplication events of *PmGAox*s, and two fragment duplication events (*PmGA20ox6* and *PmGA20ox7*, *PmGA2ox1,* and *PmGA2ox4*) were identified (Figure 3A). Collinearity relationship analysis of *P. mume* associated with *P. armeniaca*, *P. salicina*, and *P. persica* was performed, respectively (Figure 3B). Seventeen *PmGAox*s showed collinear gene pairs with *P. armeniaca*, *P. salicina,* and *P. persica*, suggesting that they evolved over a longer period of time and may have formed before species diverged. *PmGA2ox5* and *PmGA2ox9* have corresponding homologous genes in *P. persica* and *P. salicina* genome, while *PmGA3ox4* only in *P. armeniaca*. No syntenic gene pairs were discovered for *PmGA20ox1*/*4*/*8*/*9*; we surmised that some numbers in *GA20ox* were lost during the *Prunus* evolution process. Notably, some *PmGAox*s shared more than one orthologous gene pair with the three other species, such as *PmGA2ox1*/*4*/*7* and *PmGA20ox5*/*7*, which made a significant contribution the evolution of the *GAox* family in *Prunus*.

### 2.4. Cis-Element Analysis in Promoter of PmGAoxs

**Figure 4:**
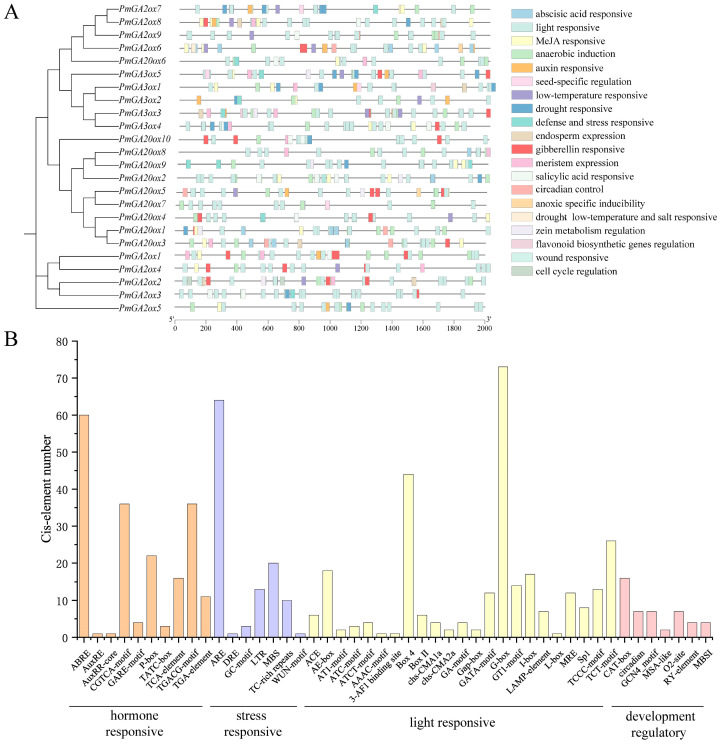
Cis-element analysis in promoter region of *PmGAox*s. (**A**) Cis-elements upstream 2 kb sequence of *PmGAox*s. Cis-elements with different functions are represented by rectangles with different colors. (**B**) The number of cis-elements in each class of *PmGAox* gene promoter region. Cis-elements are divided into hormone responsive, stress responsive, light responsive, and development regulatory classes.

Forty-seven elements predicted from the upstream 2 kb of *PmGAox*s were categorized into four categories, including hormone-responsive elements, stress-responsive elements, light-responsive elements, and development regulatory elements (Figure 4). All *PmGAox* genes have light-responsive elements, with the G-box accounting for the largest proportion and possibly having a main function in the photoresponse process. Cis-elements involved in responding to abiotic stresses such as low temperature (LTR) and drought (MBS) were discovered, thus suggesting that several *PmGAox*s may be involved in response to low temperature and/or drought conditions in *P. mume*. Ten hormone-related elements were predicted, including auxin (AuxRE, AuxRR core, TGA element), gibberellin (GARE motif, P-box, TATC box), ABA (ABRE), MeJA (CGTCA motif, TGACG motif), and salicylic acid (TCA element) responsive elements. The ABRE and MeJA responsive element had the highest number and were present in most *PmGAox*s. This suggested that ABA and MeJA may affect the endogenous GA content in plants by regulating the transcription of *PmGAox*s. In addition, a small number of development regulatory elements, such as seed specific regulation (RY-element), cell cycle regulation (MSA-like), meristem expression (CAT-box), and endosperm expression (GCN4_motif) were discovered.

### 2.5. Expression Pattern Analysis of PmGAoxs

**Figure 5:**
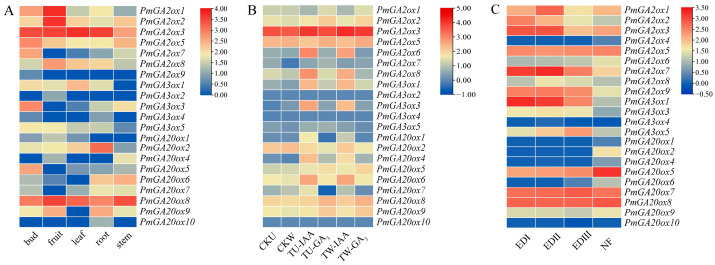
Expression profile of the *PmGAox*s. (**A**) The heatmap of expression pattern of *PmGAox*s in five tissues (bud, fruit, leaf, root and stem). (**B**) The heatmap of expression pattern of *PmGAox*s in upright and weeping stems treated with IAA or GA_3_. ‘CK’ and ‘T’ represent the control and experimental groups, respectively. ‘U’ and ‘W’ represent the straight and weeping stems of *P. mume*, respectively. ‘IAA’ and ‘GA_3_’ represent stems treated with IAA and GA_3_, respectively. (**C**) The heatmap of expression pattern of *PmGAox*s during three dormancy stages (EDI, EDII, EDIII) and natural flush (NF) stage in flower buds of *P. mume* ‘Zao Lve’.

The expression levels of *PmGAox*s exhibited considerable diversity across different organs (Figure 5A). Except for *PmGA2ox4/7*/*9*, other *PmGA2ox*s were expressed in five tissues, while in the GA20ox classes, only *PmGA20ox2*/*8* were expressed in five organs. Similarly, both *PmGA2ox3* and *PmGA20ox8* displayed high expression levels across all five tissues. The expression level of *PmGA3ox*s was generally low within the five tissues, especially in stems. In general, the expression levels of *PmGA2ox1*/*3*/*7*/*8*/*9* exhibited an upward and then downward trend, reaching the highest level at EDII stage (Endodormancy II, December, flower bud had a 45% flush rate) (Figure 5B). Most *PmGA20ox*s expression levels showed slight differences from EDI (Endodormancy I, November, flower bud had no flush sign) to NF stage (Natural Flush, February, the dormancy of flower buds had been completely released), but *PmGA20ox7* was significantly higher in the NF stage than in endo dormancy stages (Figure 5B). It is interesting that both *PmGA20ox*s and *PmGA3ox*s are involved in the GA synthesis process; the transcripts of *PmGA20ox1*/*2*/*4*/*6*/*7*/*10*/*11* were the highest at the NF stage, while most *PmGA3ox*s were the lowest at the NF stage (Figure 5B). However, *PmGA3ox5* showed an upward trend from EDI to EDII and EDIII (Endodormancy III, January, flower bud had completely flushed), indicating that *PmGA3ox* promotes GA synthesis mainly during the EDII and EDIII stage. Most *PmGAox*s exhibited lower expression levels in both straight and weeping stems (Figure 5C). However, after treatment with IAA, *PmGA2ox6*/*8*, *PmGA3ox1*/*3*, and *PmGA20ox1*/*4*/*6* were significantly upregulated in two types of stems. Moreover, some genes such as *PmGA2ox6*, *PmGA20ox5*/*8*/*9* showed an upregulation trend, while *PmGA20ox7* was significantly downregulated in upright and weeping stems under GA_3_ treatment (Figure 5C). These genes may play a crucial role in regulating GA levels in *P. mume* stem. Interestingly, some genes exhibited differential expression levels between straight and drooping stems under hormone stress. Between the upright and weeping stems treated with IAA, five genes including *PmGA2ox8*, *PmGA3ox3*, and *PmGA20ox1*/*4*/*7* were considered differentially expressed genes (DEGs), whereas only *PmGA20ox1* and *PmGA2ox8* were considered DEGs after GA_3_ treatment (Figure S2). It is worth noting that after treatment with IAA or GA_3_, *PmGA2ox8* showed significant upregulation, and differential expression was observed in the two types of stems.

### 2.6. Transcription Activation Activity of PmGA2ox8 Promoter

**Figure 6:**
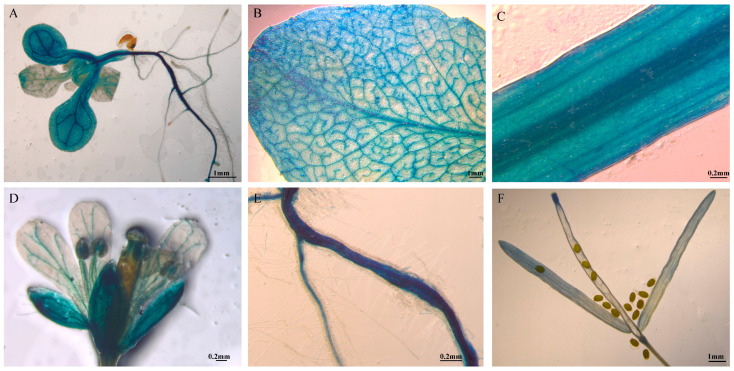
GUS staining in *PmGA2ox8_pro_*::*GUS* transgenic *Arabidopsis*. (**A**) GUS staining of transformed *Arabidopsis* in four leaf stage. (**B**–**F**) GUS staining of leaf, stem, flower, root, fruit pods, and seeds in the fruiting stage of transgenic *Arabidopsis*.

To investigate the expression pattern of *PmGA2ox8*, GUS staining was performed on *PmGA2ox8_pro_::GUS* transgenic *Arabidopsis* at different developmental stages (Figure 6). Transgenic individuals were stained in roots, stems, leaves, pods, and flower organs. GUS activity varied among different tissues, with weaker activity in lateral roots compared to main roots and stronger activity in older leaves than in younger leaves, higher in the calyx and lower in other flower tissues (Figure 6A–E). Furthermore, a relatively weak GUS activity was observed in the fruit pods, but not in the seeds (Figure 6F).

### 2.7. Overexpression of PmGA2ox8 Inhibited the Growth and Floral Development of Arabidopsis

**Figure 7:**
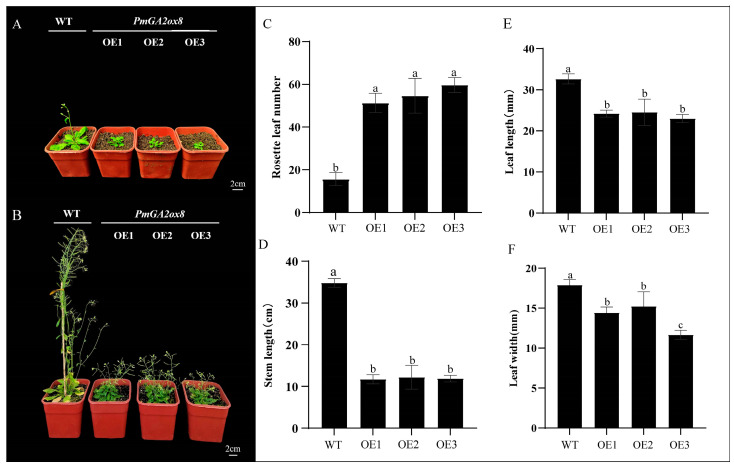
Phenotypic traits of the *PmGA2ox8* overexpressed (OE) *Arabidopsis*. (**A**) The phenotypes of *PmGA2ox8* transgenic lines and WT at 30 d. (**B**) The phenotypes of *PmGA2ox8* overexpressed lines and WT at 52 d. (**C**) The stem diameter of the *PmGA2ox8* overexpressed lines and WT. (**D**) The rosette leaf numbers of *PmGA2ox8* transgenic lines and WT at 52 d. (**E**) The leaf length of the *PmGA2ox8* overexpressed lines and WT. (**F**) The leaf width of the *PmGA2ox8* overexpressed lines and WT. Data are shown as mean ± standard deviation (SD) of three replicates. Statistical significance is based on the one-way analysis of variance (ANOVA); significant differences among means (LSD, *p* < 0.05) are represented by a, b, and c.

The coding region of *PmGA2ox8* was cloned into the pCAMBIA1301 vector and transformed into *Arabidopsis* through Agrobacterium-mediated transformation. The wild-type (WT) and *PmGA2ox8* overexpressing (OE) *Arabidopsis* lines were simultaneously sown and cultivated for phenotype identification to explore the biological function of *PmGA2ox8*. On the 30th day, compared with WT, *PmGA2ox8* OE lines exhibited smaller leaves, and WT entered the flowering period, whereas OE lines had not yet flowered (Figure 7A). During the fruiting period, significant differences were found between WT and OE lines regarding plant height, number of rosette leaves, and leaf size. The WT reached a height exceeding 30 cm, while OE lines only grew up to 10–15.4 cm (Figure 7B,C). The number of rosette leaves in WT ranged from 13 to 19, with a leaf length of 31.67–34.00 cm and a width of 17.33–18.67 cm (Figure 7D–F). The OE lines exhibited an increased number of leaves (47–63) but shorter length (21.67–28 cm) and width (11–15 cm) (Figure 7D–F).

**Figure 8:**
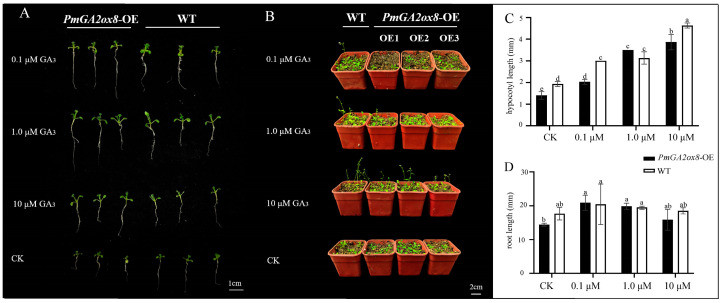
Phenotypic traits of the *PmGA2ox8* overexpressed lines and WT treated with GA_3_. (**A**) The phenotypes of *PmGA2ox8* transgenic lines and WT seedlings treated with GA_3_ or untreated at 14 d. (**B**) The phenotypes of *PmGA2ox8* transgenic lines and WT seedlings treated with GA_3_ or untreated at 21 d. (**C**) The hypocotyl length of the *PmGA2ox8* transgenic lines and WT seedlings treated with GA_3_ or untreated. (**D**) The taproot length of the *PmGA2ox8* transgenic lines and WT seedlings treated with GA_3_ or untreated. Data are shown as mean ± standard deviation (SD) of three replicates. Statistical significance is based on the one-way analysis of variance (ANOVA); significant differences among means (LSD, *p* < 0.05) are represented by a, b, c, d and e.

The 1/2 MS medium containing different concentrations of GA_3_ and without GA_3_ were used to sow *PmGA2ox8* OE lines and WT seeds, respectively. Hypocotyl length and taproot length of the seedlings were measured at 15 d (Figure 8A–C). In the control group (CK), the hypocotyl and taproot of transformed *Arabidopsis* were significantly shorter than those of WT. Under the action of GA_3_, the hypocotyl and taproot length increased, and the length of transgenic lines treated with 0.1 μM GA_3_ exceeded that of the untreated WT. As GA_3_ concentration increased, the hypocotyl length continuously increased, but root length did not show a sustained increasing trend, and no significant differences were observed between transformation and WT lines. This may be because roots are more sensitive to GA_3_ compared to the hypocotyl. Moreover, GA_3_ had the function of early flowering of *Arabidopsis*, but the transgenic lines required a higher concentration (Figure 8B). This suggested that *PmGA2ox8* may downregulate the hypocotyl and taproot length and flowering period of *Arabidopsis* by reducing endogenous active GA content. Exogenous GA_3_ can counteract the negative regulatory effect of *PmGA2ox8*.

## 3. Discussion

GAs were important hormones that affect plant growth and development. The synthesis of gibberellin mainly occurs in plant tissues with active cell proliferation, such as immature seeds and fruits, shoot tips, leaf petioles, and root tips [33]. There are differences in the distribution and function of GAs in different plants and tissues. For example, GAs are actively synthesized in the annual shoots of peach; the GA_4_ content is highest in the middle and lowest in the bottom. The GA_1_ content gradually increases from the bottom to the tip, while the GA_3_ content shows the opposite trend [19]. Although all three GAs are distributed in the stem, GA_3_ plays a greater role in plant stem elongation. The *le* pea mutant lacking GA_1_ in the stem showed dwarfism, indicating that GA_1_ acts on pea stem elongation [34]. However, the internode elongation of poplar was related to the GA_4_ level, but not to GA_1_ [21]. The regulation of endogenous GAs in plants was controlled by GAox enzymes, with GA20ox and GA3ox controlling GA synthesis, while GA2ox regulates GA metabolism. Our study conducted genome-wide identification of the GAox family in four *Prunus* species. There were 24, 18, 22, and 21 *GAox* genes in *P. mume*, *P. armeniaca*, *P. salicina,* and *P. persica* genome, respectively. A phylogenetic tree was constructed based on the GAox protein sequences of four *Prunus* species, Arabidopsis, and rice. All GAoxs were classified into six categories, referring to previous classifications of the rice GAox family [35]. Most GAoxs in *Prunus* could be clustered together with GAoxs in rice and *Arabidopsis*, possibly because they may share a common ancestor. However, PmGA2ox5 and PsGA2ox3 had the longest clades, with no closely related genes in *P. persica*, *P. armeniaca*, *Arabidopsis*, and rice. This may indicate that although *P. mume* is the latest to differentiate in *Prunus* species, it has some ancient genes that were not involved in differentiation in the evolution of some *Prunus* species [36]. Similarities in structure and motif distribution among closely related genes indicated that *GAox*s were highly conserved among the four *Prunus* species. Among the 10 predicted motifs, 8 are shared by almost all GAoxs, especially Motifs 1 and 2, which belong to 2OG-FeIIoxygenase and DIOX_N domains, respectively. These two domains were conserved in the GAox family that regulate endogenous GAs content in plants. The specific presence or absence of motif 8 and motif 10 in a certain subfamily may contribute to differences in gene function. Two fragment replication events were discovered in the *PmGAox* family, which may be attributed to the fact that only one replication event has occurred in the evolutionary history of the *P. mume* genome [36]. Collinearity analysis revealed that most *PmGAox*s had homologous genes in *P. armeniaca*, *P. salicina,* and *P. persica*, due to the close genetic relationship between *P. mume* and the three other *Prunus* species.

Light-responsive elements, multiple hormone-responsive elements, abiotic stress-responsive elements, and development regulatory elements were found in the *PmGAox*s promoter region, similar to those found in plants such as apple, grape, and *Liriodendron chinense* [15,16,17]. Previous studies found that the expression levels of *GAox*s were influenced by factors such as hormones, temperature, and light. For example, after 100 mg/L GA_3_ treatment of peach, the expressions of *PpGA2ox1*/*2*/*4*/*5*/*6* increased significantly after 2–4 h; *PpGA2ox3* was first downregulated and then up-regulated [19]. The expression levels of *VvGAox*s showed significant differences in grape treated with different concentrations of GA_3_ [16]. In our study, transcriptome sequencing data of *P. mume* stems treated with GA_3_ for 6 h were used to analyze the expression levels of *PmGAox*s, and it was found that the expression levels of most *PmGAox*s changed slightly. This may be due to the low concentration of GA used, only 2 mg/L. It is speculated that exogenous GA regulates the transcription levels of *PmGAox*s in a dose-dependent manner. Although *PmGAox*s are gibberellin oxidase genes, not all genes were found to possess GA-responsive elements. This may be due to the regulation of GA by these genes through other factors on the GA signaling pathway, or the presence of GA-related elements beyond 2 kb upstream of the start codon. IAA responsive elements were discovered in *PmGAox* promoters, and transcriptome analysis results showed that exogenous IAA can affect the expression of *PmGAox*s, which were confirmed in species such as Arabidopsis and peas [37,38]. In addition, previous studies have shown that *GAox* expression levels in many plants such as Arabidopsis, rice, and pea were regulated by ABA, low temperature, drought, and salt [23]. Significant changes were observed in the expression levels of *HvGA2ox1*/*3*/*6* in the coleoptile and root of barley seedlings treated with NaCl [39]. After low-temperature treatment, *LcGA2ox1* and *LcGA2ox4* were upregulated in the roots, stems, and leaves of *L. mandshurica* [17]. ABA, low temperature, and drought-responsive elements were found in *PmGAox* promoters, suggesting that *PmGAox*s could respond to ABA, drought, and low temperature. However, only one DRE element associated with salt stress was found in the *PmGA2ox6* promoter. Whether *PmGAox*s can respond to salt stress and through which pathway needs further investigated.

Many studies have shown that *GAox*s expression in various species was tissue-specific. For example, *BnaGA2ox2c*/*6b*/*6d* were preferentially expressed in flower organs [40]. *CsGA20ox1* was significantly expressed in the root and had a function of promoting taproot and lateral root development [41]. Our study found specificity in the expression of *PmGAox*s. For example, *PmGA20ox10* was only expressed in roots, while *PmGA20ox2* was expressed in roots more than in other organs. The transcription levels of *PmGA2ox1*/*2* were significantly higher in fruit than in root, stem leaf, and buds. Interestingly, the transcription levels of several *PmGAox*s showed obvious differences between upright and weeping stems of *P. mume*. Almost all *PmGA2oxs*, especially *PmGA2ox7*/*8,* were expressed at higher levels in straight stems than in vertical stems, while *PmGA3ox3*, *PmGA20ox7*/*9* were expressed at higher levels in drooping stems than in straight stems. Our previous study found that in mature annual stems of *P. mume*, the content of GA in weeping stems was higher than that in straight stems, which was probably caused by the difference in *GAox*s expression [42]. GA was believed to be related to the early development of the weeping trait of crape myrtle. *LifGA2ox* was found to be differentially expressed in upright and weeping stems of crape myrtle, with higher expression in weeping trees than in upright trees in several oranges (including axillary shoot, stem, axillary bud and leaf) [43]. This is contrary to our results, possibly due to different plants having varying sensitivities to GAs. It may also be due to differences in sampling time, as the expression of *GAox*s varies during different developmental stages of plant tissues. GA has the function of stimulating stem cell elongation and division to affect plant type. IAA can regulate GA level by regulating the expression of specific *GAox*. The promotion of GA synthesis or metabolism by IAA depends on different tissues, resulting in different responses [44]. For example, IAA may upregulate *AtGA2ox2* at the boundary of meristem tissue to inactivate GA, thereby participating in maintaining the activity of stem tip meristem tissue, or upregulate *AtGA20ox2* to increase GA level and promote cotyledon growth [45]. Due to the significant increase in *PmGA2ox8* expression in stems treated with IAA or GA_3_, it is speculated that *PmGA2ox8* plays a key role in responding to GA_3_ and IAA to regulate GA level in *P. mume* stems. In addition, *PmGA2ox8* displayed differential expression in straight and weeping stems of *P. mume* that were untreated and treated with IAA or GA_3_. A previous study observed that straight and weeping stems displayed varying degrees of bending under the action of GA_3_ and IAA [46]. We speculated that *PmGA2ox8* is pivotal to stem development and plant type formation of *P. mume*. GAoxs may be related to the formation of plant architecture in *Lagerstroemia indica*. The transcript levels of *LiGA3ox2/5/6*, *LiGA20ox5*, and *LiGA2ox9/19/20* were higher in the lower tissues than in the upper tissues of the curved part of branches, while the mRNA levels of *LiGA3ox1*, *LiGA20ox4*, and *LiGA2ox8/13/16* were higher in the upper tissues [47]. The expression patterns of *PmGAox* genes differed among the developmental stages in winter of flower buds. From EDI to NF stage, the GAs (GA_1_, GA_3_, GA_4_) content in flower buds of *P. mume* first increased and then decreased, reaching its lowest point in the EDII stage and the highest point in the NF stage [48]. The changes in GAs level may be attributed to the transcriptional enrichment of *PmGAox*s. The expression levels of *PmGA2ox1*/*3*/*7*/*8*/*9* were upregulated from the EDI to EDII stages and then sharply downregulated to the EDIII and NF stages. Most *PmGA3ox*s exhibited the lowest transcription level at the NF stage, but *PmGA3ox5* expression level significantly increased from EDI to EDII and EDIII. Although the expression levels of *PmGA20ox1*/*2*/*4*/*6*/*7*/*10*/*11* were highest in the NF stage, most of them showed weak changes during the four stages. However, the expression level of *PmGA20ox7* increased sharply from the EDIII to NF stage. In summary, *PmGAox*s played an important role in regulating bud dormancy of *P. mume*. During the development stage of fruits of tomato, *SlGA2oxs* exhibited different expression patterns. The transcript level of *SlGA2ox1* was higher in immature green fruits than in mature green fruits and red ripening fruits, but *SlGA2ox2/4/5* were higher in fruits at the mature green stage. *SlGA2ox8* is mainly expressed in red ripening fruits and is almost not expressed in immature and mature green fruits [49].

*PmGA2ox8* was 891 bp in length and contained four exons located on chromosome 2 of *P. mume*. Phylogenetic analysis showed that *PmGA2ox8* co-clustered with genes such as *OsGA2ox5*/*6*/*9*/*11*, *AtGA2ox7*/*8*, *PaGA2ox3*, *PsGA2ox2*, and *PpGA2ox2* in the C20-GA2ox subfamily. Therefore, *PmGA2ox8* may have the function of encoding C20-GA2ox. The collinearity analysis showed that *PmGA2ox8* had collinearity with *PaGA2ox3*, *PsGA2ox2,* and *PpGA2ox2*. Seven conserved motifs were distributed on the PmGA2ox8 protein sequence, among which motif 1 belonged to the 2OG-FeIIoxygenase domain, which is a conserved domain of the *GAox* family. Photoresponsive elements, hormone (gibberellin, auxin, MeJA, and salicylic acid) and abiotic stress (low temperature, drought, anaerobic induction) response elements, and plant development regulatory elements involved in meristem and endosperm expression were found in the *PmGA2ox8* promoter. In our study, the *PmGA2ox8* promoter sequence was cloned and linked to pCAMBIA1301 vector to transform *Arabidopsis*. GUS staining displayed that the *PmGA2ox8* promoter could drive *GUS* reporter gene expression in taproots, lateral roots, stems, leaves, and flower organs of *Arabidopsis* with different expression intensities, indicating that the expression of *PmGA2ox8* was spatio-temporal specific. The coding sequence of *PmGA2ox8* was cloned from the *P. mume* genome and transferred into *Arabidopsis*. The *PmGA2ox8* overexpressed lines showed dwarfism, indicating that *PmGA2ox8* can negatively regulate plant height. This is consistent with results in a variety of plants, such as rice, pear, sweet potatoes, and peach [19,22,50]. Overexpression of *PmGA2ox8* upregulated the rosette leaves number but downregulated the leaf area and delayed the flowering of *Arabidopsis*. According to the previous study, the upregulation of *AtGA2ox7* and *AtGA2ox8* induced the decrease of endogenous active GA in plants, resulting in dwarfing, delayed flowering, and increased number of leaves [31]. The GA_1_ and GA_4_ content in cultivated soybean (with shorter shoot length) was lower than in wide soybean (with longer shoot length), and the transcription level of *GmGA2ox8* was higher in cultivated soybean. *GmGA2ox8* overexpressed soybean showed a phenotype with dwarfing, shorter roots, and smaller leaf area due to the decrease in active GA content, while knockdown and knockout of *GmGA2ox8* increased shoot and internode length in soybeans [32]. Cheng et al. found that *PpGA2ox1*, *PpGA2ox5*, and *PpGA2ox2* in pear genome could reduce plant height and leaf area of tobacco. Similar to *PmGA2ox8*, *PpGA2ox2* belongs to the C20-GA2ox subfamily. The level of GA_4_ were lower in *PpGA2ox2* overexpressed tobacco than in WT [19]. In our study, the hypocotyl and taproot length of *PmGA2ox8* overexpressed *Arabidopsis* seedlings were shorter than those of WT, but increased significantly after GA_3_ treatment. In the same way, overexpression of *ClGA2ox3* gene from *Camellia lipoensis* can lead to a decrease in the endogenous activities GA_1_ and GA_4_ in *Nicotiana tabacum*, resulting in reduced plant height, smaller leaves, and delayed flowering. This dwarfing phenotype was rescued by the application of GA_3_ [51]. Therefore, we speculated that *PmGA2ox8* can inactivate the endogenous active GAs in plants and affect the formation of plant architecture. In addition, the germination rate of *PavGA2ox-2L* overexpressed *Arabidopsis* seeds was lower than that of wild-type, and the seed germination of *OsGA2ox9* overexpressed lines of rice was inhibited [21,52]. However, the seed germination rate of *Arabidopsis* overexpressed with *PmGA2ox8* was consistent with that of wild-type. Similarly, leaf development of *Arabidopsis* was not affected by *PavGA2ox-2L* [21]. These results indicated that the effects of *GA2ox*s on leaf development and seed dormancy in different species were varied.

## 4. Materials and Methods

### 4.1. Plant Material and Growth Conditions

Upright and weeping individuals of F1 population of *P. mume* ‘Liuban’ × ‘Fentai Chuizhi’ were grown in a greenhouse of Beijing Forestry University. Mature annual stems of upright and drooping individuals were collected and rapidly frozen with liquid nitrogen, followed by storage at −80 °C for subsequent RNA extraction.

Seeds of wild-type and transgenic lines of *Arabidopsis* were sown in Murashige and Skoog (MS) medium (Beijing XMJ Scientific Co., Ltd., Beijing, China) containing 0.7% (*w*/*v*) agar (Biorigin Co., Ltd., Beijing, China), 30% (*w*/*v*) sucrose (Biorigin Co., Ltd., Beijing, China) and placed at 4 °C for vernalization and then cultured under conditions of 22 ± 2 °C, 16 h/8 h (light/dark) and 80% relative humidity. After 7 d, the seedlings were transplanted into a square pot containing substrate (peat:vermiculite:perlite = 1:1:1) and grown under 16–25 °C, 12 h light/dark cycle, and 60% relative humidity conditions in the artificial climate chamber.

### 4.2. Identification and Physicochemical Properties Analysis of GAox Genes in Four Prunus Species

*Prunus mume* genome data were download from the Rosaceae genome database GDR (available online: https://www.rosaceae.org/, accessed on 28 September 2023), while the predicted genome data of *P. armeniaca*, *P. salicina*, *P. persica*, *A. thaliana,* and *Oryza sativa* were acquired from the Ensembl Plants (available online: https://plants.ensembl.org, accessed on 28 September 2023) [53]. Sixteen *Arabidopsis* GA2ox, GA3ox, and GA20ox protein sequences were downloaded from the TAIR website (available online: http://www.arabidopsis.org/, accessed on accessed on 28 September 2023) [54] for querying sequences [55]. The TB tools-II (Toolbox for Biologists) v2.096 software’s BLAST project was utilized to obtain the GAox prediction protein sequences, followed by hidden Markov model (HMM) searches [56] to eliminate amino acid sequences lacking the 2OG-Fell_Oxy (PF03171) and DIOX_ N (PF14226) domains (available online: http://pfam.janelia.org, accessed on 28 September 2023). After removing redundant and repetitive sequences, gene annotation information from the NCBI website (available online: https://www.ncbi.nlm.nih.gov/, accessed on 2 October 2023) was applied to identify homologous GAox sequences. Subsequently, we conducted an evaluation of physicochemical properties, including amino acids number (aa), isoelectric point (*pI*), and molecular weight (MW), utilizing the ExPASy website (available online: http://web.expasy.org/protparam/, accessed on 10 October 2023); meanwhile, subcellular localization was assessed through CELLOGO (available online: http://cello.life.nctu.edu.tw/, accessed on 10 October 2023).

### 4.3. Phylogenetic and Evolution Analysis

Phylogenetic analysis was performed on GAoxs sequences identified from four *Prunus* species (*P. mume*, *P. armeniaca*, *P. persica*, and *P. salicina*), along with 16 *Arabidopsis* and 19 rice GAoxs [35,57]. Molecular Evolutionary Analysis (MEGA X) version 10.0.5 software was used to align all GAox sequences and then construct the phylogenetic tree with the Neighbor-Joining (NJ) method. Eventually, the phylogenetic tree was beautified by ChiPlot (available online: https://www.chiplot.online/, accessed on 15 October 2023) [58].

### 4.4. Gene Structure and Protein Motif Identification Analysis

The MEME program (available online: https://meme-suite.org/meme/tools/meme, accessed on 20 October 2023) [59] was utilized to predict conserved motifs of GAox in four *Prunus* species, with a set number of 10 motifs. TB tools facilitated visualization of intron/exon structure of *GAox* genes as well as conserved motifs of GAox proteins [60].

### 4.5. Syntenic and Cis-Element Analysis of PmGAoxs

We used the Multiple Collinearity Scan Toolkit (MCScanX) in TB tools to analyze the repetitive events within *P. mume* genome, and orthologous genes between *P. mume* and three other *Prunus* species (*P. armeniaca*, *P. salicina*, and *P. persica*) [60]. The upstream 2 kb sequence of the transcriptional start site of *PmGAox*s served as promoter region for identifying cis-regulatory elements via PlantCARE online database (available online: (http://bioinformatics.psb.ugent.be/webtools/plantcare/html/, accessed on 14 November 2023).

### 4.6. Expression Patterns of PmGAoxs

To reveal the expression patterns of *PmGAox*s in endo dormancy stages, different tissues and response to hormone stress, three sets of raw data from RNA-seq were employed: (1) Five tissues including flower buds, leaves, stems, fruits, and roots of *P. mume* [61]. (2) Buds of *P. mume* ‘Zao Lve’ (growing on the open field in Beijing) undergoing four developmental stages in winter: EDI (Endodormancy I, November, flower bud had no flush sign), EDII (Endodormancy II, December, flower bud had a 45% flush rate), EDIII (Endodormancy III, January, flower bud had completely flushed), and NF stage (Natural Flush, February, the dormancy of flower buds had been completely released) [48]. (3) The upright and weeping stems after being treated with water, IAA, or GA_3_ for 6 h, which were collected from the F_1_ separate populations of *P. mume* ‘Liuban’ × ‘Fentai Chuizhi’ (Figure S1) [46]. Excel was used to analyze data, and TB tools was used to draw heat maps.

### 4.7. Cloning, Vector Construction, and Plants Transformation

The coding sequence (CDS) and promoter fragments (upstream 2 kb sequence) of *PmGA2ox8* were amplified from the cDNA of weeping individuals in the F_1_ generation of ‘Liuban’ × ‘Fentai Chuizhi’ with specific primers (Table S2). CDS and promoter of *PmGA2ox8* were connected to the pCAMBIA1301 and pCAMBIA1300 vector, respectively, resulting in the overexpression vectors (pCAMBIA1301-*PmGA2ox8* and pCAMBIA1300-*PmGA2ox8_pro_*::*GUS*). The pCAMBIA1301-*PmGA2ox8* and pCAMBIA1300-*PmGA2ox8_pro_*::*GUS* recombinant vector were independently transferred into *Agrobacterium tumefaciens* strain GV3101 (Shanghai Weidi Biotechnology, Shanghai, China) for plant transformation. The inflorescence impregnation method was used to transform *Arabidopsis*. The screening medium (1/2 MS + 50 mg/L hygromycin B) was used to select the resistant lines. Positive lines were verified by PCR detection and qRT-PCR analysis (Figure S2). Specific primers were listed in Table S2.

### 4.8. GUS Histological Staining

GUS staining solution was obtained from Biorigin Co., Ltd. (Beijing, China) X-gluc (50×) and buffer was mixed in a 1:50 ratio to prepare the working solution, which was added to a centrifuge tube to completely cover the *PmGA2ox8_pro_::GUS* transgenic *Arabidopsis*. it was stained overnight at 37 °C for staining, and then washed with 75% alcohol and replaced with alcohol multiple times until all chlorophyll in the plant tissue degraded.

### 4.9. Exogenous GA_3_ Treatment

Seeds of wild-type and *PmGA2ox8* overexpression *Arabidopsis* were sown on 1/2 MS medium (Beijing XMJ Scientific Co., Ltd, Beijing, China) containing 0.7% (*w*/*v*) agar, 30% (*w*/*v*) sucrose, and different concentrations (0.1 μM, 1 μM, and 10 μM) of GA3 or GA3 free and placed at 4 °C for 3 d for vernalization, and then cultured at 22 ± 2 °C 16/8 h (day/night) with 80% humidity. After 15 d, the lengths of hypocotyl and taproot of seedlings were measured. The seedlings were then transplanted into a square pot (with a side length of 10 cm, each pot containing four seedings) and placed in the greenhouse (16–25 °C, 12 h light/dark cycle, and 60% relative humidity) for cultivation. Seven days later, the seedlings were sprayed with GA3 at the same concentration as in the 1/2 MS medium.

### 4.10. Statistical Analysis

All data from wild-type and *PmGA2ox8* overexpressed *Arabidopsis* were repeated three times to calculate the mean ± standard deviation. One-way analysis of variance (ANOVA) was performed to compare the mean values. Fisher’s least significant difference test (LSD, *p* < 0.05) was employed to determine the mean values difference between individuals.

## 5. Conclusions

Eighty-five *GAox* genes were identified in the genomes of four *Prunus* species and divided into six subgroups. GAoxs among the four species exhibited high conservatism and there were many gene pairs between *P. mume* with the three other species. Hormone-responsive elements, stress-responsive elements, light-responsive elements, and development regulatory elements were found in the *PmGAox*s promoter region. The *PmGAox*s exhibited specific expression patterns in endo dormancy stages, different organs, and response to hormone stress. Furthermore, overexpression of *PmGA2ox8* in Arabidopsis reduced the plant height and leaf area, but increased the number of rosette leaves and delayed flowering.

## Data Availability

The data presented in this study are available on request from the corresponding author.

## Supplementary Materials

The following supporting information can be downloaded at: https://www.mdpi.com/article/10.3390/ijms25168697/s1.
