# Supplementary material for: Genome-Wide Analysis of the Gibberellin-Oxidases Family Members in Four Prunus Species and a Functional Analysis of PmGA2ox8 in Plant Height
Source: Int J Mol Sci. 2024 Aug 9;25(16):8697. doi: 10.3390/ijms25168697 (PMC11354515; doi:10.3390/ijms25168697)
Supplement: Supplementary file 1 [file ijms-25-08697-s001.zip › ijms-3115528-supplementary.pdf]

Table S1. The identification and characteristics of GAoxs in four Prunus species

species	Gene ID	Gene name	Chromosome Localization	CDS (bp)	aa	MW (Da)	pI	Subcellular Localization
	Pm008423	*PmGA2ox1*	chr2:33021509:33024861	1014	390	44387.49	6.23	Cytoplasm
	Pm010412	*PmGA2ox2*	chr3:4308416-4309913	1026	333	37482.8	6.97	Cytoplasm
	Pm011163	*PmGA2ox3*	chr3:9358924-9360357	1002	354	39487.99	6.76	Cytoplasm
	Pm012916	*PmGA2ox4*	chr4:439344-442395	1065	372	41017.72	7.2	Cytoplasm
	Pm018939	*PmGA2ox5*	chr5:21351170-21352492	1137	372	42135.73	5.78	Cytoplasm
	Pm000588	*PmGA2ox6*	chr1:3606425-3608033	1005	337	37277.41	6.01	Cytoplasm
	Pm009744	*PmGA2ox7*	chr3:725715-730306	1173	341	37983.75	5.62	Cytoplasm
	Pm005983	*PmGA2ox8*	chr2:14086171-14087789	891	384	43407.88	5.82	Cytoplasm
	Pm029487	*PmGA2ox9*	scaffold265:562255-565535	1056	296	33111.74	5.73	Cytoplasm
	Pm004966	*PmGA3ox1*	chr2:8376988-8378362	1035	343	37950.59	5.69	Cytoplasm
	Pm004968	*PmGA3ox2*	chr2:8397997-8399436	1032	387	43598.64	6.46	Cytoplasm
Prunus	Pm013603	*PmGA3ox3*	chr4:5317568-5319039	1119	356	39231.86	5.84	Cytoplasm
mume	Pm016892	*PmGA3ox4*	chr5:5674202-5675581	1071	374	42676.46	6.32	Cytoplasm
	Pm027461	*PmGA3ox5*	chr8:15353488-15355882	1017	351	39942.4	6.47	Cytoplasm
	Pm004375	*PmGA20ox1*	chr2:4477260-4478775	1155	384	43406.9	5.91	Cytoplasm
	Pm004807	*PmGA20ox2*	chr2:7285321-7286604	1065	354	40554.98	5.38	Cytoplasm
	Pm005214	*PmGA20ox3*	chr2:9841897-9843184	663	220	24878.55	4.8	Cytoplasm
	Pm005215	*PmGA20ox4*	chr2:9847015-9849218	1164	334	37410.5	6.15	Cytoplasm
	Pm018083	*PmGA20ox5*	chr5:16081097-16082600	1125	378	41402.55	5.3	Cytoplasm
	Pm018147	*PmGA20ox6*	chr5:16488978-16490234	987	328	37284.41	5.55	Cytoplasm
	Pm019411	*PmGA20ox7*	chr5:23765236-23766540	1119	358	40270.29	5.68	Cytoplasm
	Pm021552	*PmGA20ox8*	chr6:9598551-9600074	1071	356	40992.66	5.63	Cytoplasm
	Pm021553	*PmGA20ox9*	chr6:9600674-9602197	1065	354	40839.27	5.94	Cytoplasm
	Pm023365	*PmGA20ox10*	chr7:4436245-4437852	1077	338	38009.77	8.58	Cytoplasm
	PARG00505m02	*PaGA2ox1*	chr1:3372873-3374720	1005	334	37399.42	5.87	Cytoplasm
	PARG04470m03	*PaGA2ox2*	chr2:9105442-9107604	861	286	31494.84	5.16	Cytoplasm
	PARG06969m01	*PaGA2ox3*	chr2:27340772-27343667	1035	344	38823.26	6.37	Cytoplasm
	PARG10473m01	*PaGA2ox4*	chr3:10428868-10434851	1143	380	43108.00	7.10	Cytoplasm
	PARG11108m01	*PaGA2ox5*	chr3:15468848-15470953	1002	333	37482.80	6.97	Cytoplasm
	PARG11902m02	*PaGA2ox6*	chr3:20797411-20798935	1026	341	37984.73	5.49	Cytoplasm
	PARG16163m01	*PaGA2ox7*	chr4:25373992-25377235	1059	352	39259.79	6.76	Cytoplasm
	PARG08067m01	*PaGA3ox1*	chr2:34111292-34120541	1305	434	47926.14	6.74	Cytoplasm
Prunus	PARG08069m01	*PaGA3ox2*	chr2:34139702-34141131	1032	343	37994.60	5.59	Cytoplasm
apricot	PARG15458m01	*PaGA3ox3*	chr4:19796977-19798656	1119	372	41053.80	7.73	Cytoplasm
	PARG17349m01	*PaGA3ox4*	chr5:10101436-10102999	1071	356	39271.86	5.84	Cytoplasm
	PARG27977m01	*PaGA3ox5*	chr8:20068948-20070968	1023	340	38404.21	8.59	Cytoplasm
	PARG07860m01	*PaGA20ox1*	chr2:32739326-32741543	1164	387	43565.56	6.36	Cytoplasm
	PARG08726m01	*PaGA20ox2*	chr2:38787217-38788728	1155	384	43526.16	5.97	Cytoplasm
	PARG18139m01	*PaGA20ox3*	chr5:16365354-16366864	1134	377	42991.87	6.32	Cytoplasm
	PARG19406m02	*PaGA20ox4*	chr5:24495277-24496415	918	305	34280.69	5.11	Cytoplasm
	PARG23462m01	*PaGA20ox5*	chr7:6760487-6762083	1077	358	40210.22	5.81	Cytoplasm
	PARG29952m01	*PaGA20ox6*	tig00008438:133384-135017	987	328	37314.52	5.70	Cytoplasm

Prunus persica	transcript:ONI27933	*PpGA2ox1*	chr1:8938879-8942671	1014	337	37253.32	6.01	Cytoplasm
	transcript:ONI32002	*PpGA2ox2*	chr1:32238978-32241847	1035	344	38753.19	6.08	Cytoplasm
	transcript:ONI14758	*PpGA2ox3*	chr3:403444-407130	1056	351	39121.7	6.71	Cytoplasm
	transcript:ONI10090	*PpGA2ox4*	chr4:1228553-1231453	1047	348	39382.94	6.16	Cytoplasm
	transcript:ONI10990	*PpGA2ox5*	chr4:3917455-3919603	1026	341	38046.81	5.76	Cytoplasm
	transcript:ONI12197	*PpGA2ox6*	chr4:8592732-8594638	1002	333	37546.85	7.01	Cytoplasm
	transcript:ONI13130	*PpGA2ox7*	chr4:12734733-12738568	987	328	37600.77	6.72	Cytoplasm
	transcript:ONH99879	*PpGA2ox8*	chr6:3909544-3911429	1005	334	37250.23	5.74	Cytoplasm
	transcript:ONI34192	*PpGA3ox1*	chr1:38957960-38960572	996	331	36526.93	6.14	Cytoplasm
	transcript:ONI34195	*PpGA3ox2*	chr1:38964976-38966490	1032	343	38060.62	5.78	Cytoplasm
	transcript:ONI21373	*PpGA3ox3*	chr2:7889552-7890932	1071	355	39034.5	5.72	Cytoplasm
	transcript:ONI16046	*PpGA3ox4*	chr3:5551077-5553011	1119	372	41046.78	7.72	Cytoplasm
	transcript:ONH98202	*PpGA3ox5*	chr7:20464205-20466407	1026	341	38452.39	8.3	Cytoplasm
	transcript:ONI33706	*PpGA20ox1*	chr1:37527826-37530411	1164	387	43550.61	6.23	Cytoplasm
	transcript:ONI33707	*PpGA20ox2*	chr1:37532722-37534887	726	241	27382.86	8.66	Cytoplasm
	transcript:ONI35429	*PpGA20ox3*	chr1:43765839-43767321	1155	384	43489.06	5.87	Cytoplasm
	transcript:ONI35435	*PpGA20ox4*	chr1:43804042-43805326	957	319	35771.55	5.76	Cytoplasm
	transcript:ONI22778	*PpGA20ox5*	chr2:20565140-20569734	1134	377	42891.83	6.19	Cytoplasm
	transcript:ONI23099	*PpGA20ox6*	chr2:21743171-21744738	987	328	37288.44	5.66	Cytoplasm
	transcript:ONI25182	*PpGA20ox7*	chr2:28275407-28277374	1164	387	43844.8	6.13	Cytoplasm
	transcript:ONI06440	*PpGA20ox8*	chr5:6525308-6527252	1077	358	40344.45	6.1	Cytoplasm
Prunus salicina	evm.model.LG01.4717	*PsGA2ox1*	chr1:38033030-38036332	1014	337	37426.49	5.84	Cytoplasm
	evm.model.LG01.1352	*PsGA2ox2*	chr1:11531023-11532681	1035	344	38807.26	6.08	Cytoplasm
	evm.model.LG02.805	*PsGA2ox3*	chr2:4166210-4167482	1158	385	42530.56	4.88	Cytoplasm
	evm.model.LG03.1448	*PsGA2ox4*	chr3:16105178-16110654	1161	386	43391.13	6.21	Cytoplasm
	evm.model.LG03.1528	*PsGA2ox5*	chr3:16750174-16753381	1062	353	39368.78	6.76	Cytoplasm
	evm.model.LG04.1422	*PsGA2ox6*	chr4:10216306-10217735	1002	333	37516.78	7.01	Cytoplasm
	evm.model.LG04.1917	*PsGA2ox7*	chr4:15063966-15070283	1071	356	40568.11	6.47	Cytoplasm
	evm.model.LG04.256	*PsGA2ox8*	chr4:1247534-1257312	2163	720	83095.71	8.42	Cytoplasm
	evm.model.LG04.777	*PsGA2ox9*	chr4:4494831-4496355	1026	341	38066.82	5.77	Cytoplasm
	evm.model.LG06.2494	*PsGA2ox10*	chr6:21078208-21079814	1005	334	37297.25	5.87	Cytoplasm
	evm.model.LG01.2496	*PsGA3ox1*	chr1:18553756-18555199	1032	343	37886.46	5.69	Cytoplasm
	evm.model.LG02.2609	*PsGA3ox2*	chr2:30769341-30773492	1164	387	42729.92	6.15	Cytoplasm
	evm.model.LG03.707	*PsGA3ox3*	chr3:9343674-9345146	1119	372	41020.72	6.83	Cytoplasm
	evm.model.LG07.360	*PsGA3ox4*	chr7:1870998-1873230	1026	341	38402.23	8.79	Cytoplasm
	evm.model.LG01.2268	*PsGA20ox1*	chr1:17063141-17065348	1164	387	43551.58	6.36	Cytoplasm
	evm.model.LG01.2269	*PsGA20ox2*	chr1:17069898 17071214	771	256	28686.46	8.51	Cytoplasm
	evm.model.LG01.3111	*PsGA20ox3*	chr1:23154580-23156074	1155	384	43515.14	6.06	Cytoplasm
	evm.model.LG02.1544	*PsGA20ox4*	chr2:9868069-9869311	987	328	37258.41	5.66	Cytoplasm
	evm.model.LG02.1683	*PsGA20ox5*	chr2:11059028-11060539	1134	377	43019.94	6.32	Cytoplasm
	evm.model.LG02.296	*PsGA20ox6*	chr2:1532664-1534011	1164	387	43721.66	5.78	Cytoplasm
	evm.model.LG05.566	*PsGA20ox7*	chr5:6513806-6515425	1077	358	40297.37	5.88	Cytoplasm
	evm.model.Contig38.4	*PsGA20ox8*	Coting38:20455-21949	1155	384	43515.14	6.06	Cytoplasm

Table S2. The primer sequences used in this study

Primer name	Primer sequence (5'- 3')	Purpose
PmGA2ox8-F	ATGATAGAATCAAACCCACCTC	*PmGA2ox8* coding region cloning
PmGA2ox8-R	TTATTGAAGAAGAAATCTTGAGAGG	
PmGA2ox8pro-F	GCCGCCTGATTGGTCCCTTGC	*PmGA2ox8* promoter cloning
PmGA2ox8pro-R	ACAAAGAGAGTCCAAGGTTGCGTCA	
PmGA2ox8-RT-F	AGAGAAAAGCTACTTGTGGGC	Expression levels analysis of *PmGA2ox8* in the *Arabidopsis* lines
PmGA2ox8-RT-R	TCCGAGATTTTGGTGACAGG	
AtActin-RT-F	GGTGATGGTGTGTCTCACACTG	
AtActin-RT-R	GAGGTTTCCATCTCCTGCTCGTAG	
NPT-RT-F	AAGATGGATTGCACGCAGGT	Positive lines detection of transgenic Arabidopsis
NPT-RT-R	TCACGGGTAGCCAACGCT	
HPT-RT-F	GGTCGCGGAGGCTATGGATGC	Positive lines detection of transgenic Arabidopsis
HPT-RT-R	GCTTCTGCGGGCGATTTGTGT	

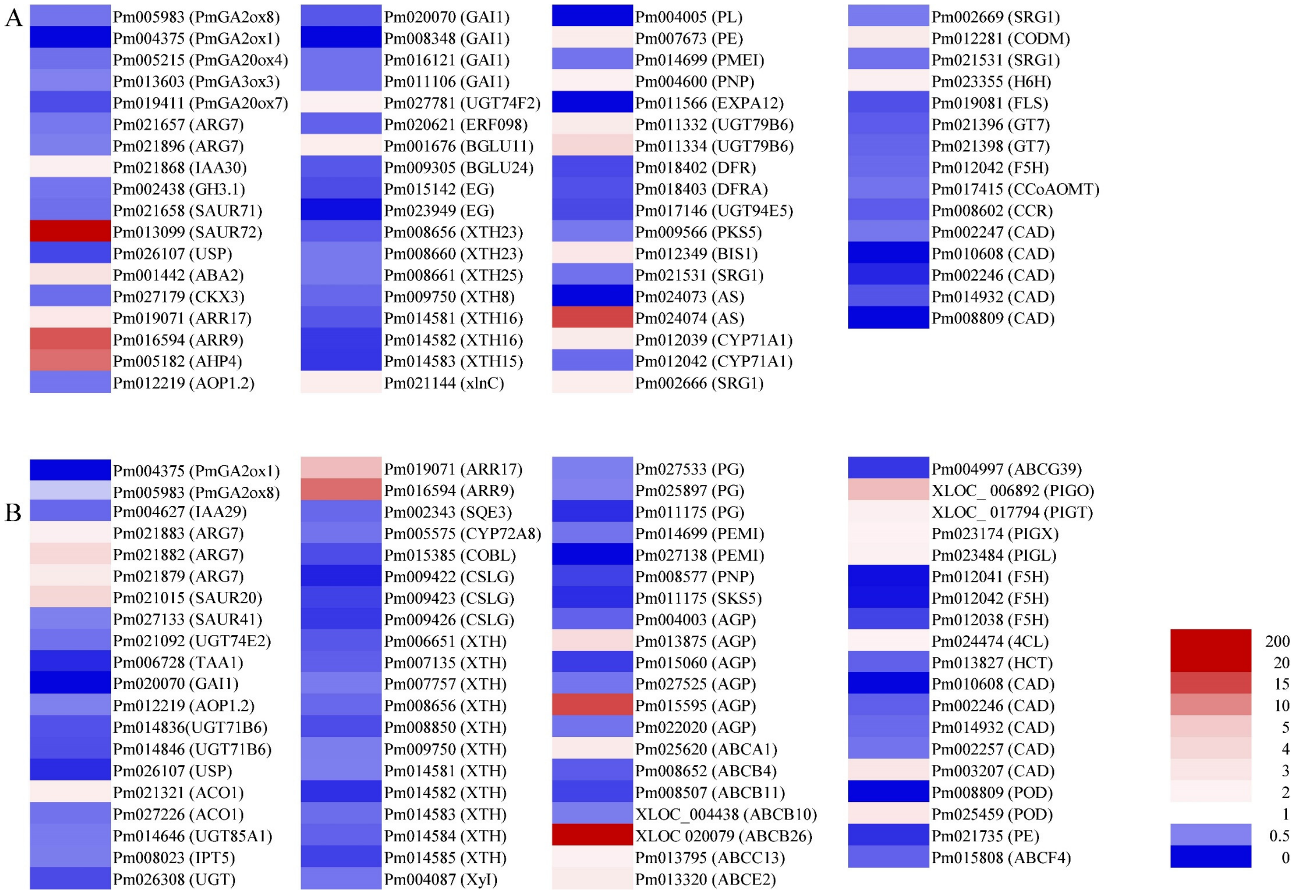

Fig S1. Clustering of DEGs in W_{IAA} vs. U_{IAA} and W_{GA} vs. U_{GA}. (A) DEGs in W_{IAA} vs. U_{IAA}. (B) DEGs in W_{GA} vs. U_{GA}. Red and blue indicate up- and down-regulated genes, respectively (fold change). *ARG*, *indole-3-acetic acid-induced protein*; *GH3.1*, *indole-3-acetic acid-amido synthetase*; *SAUR*, *auxin-responsive protein*; *UGT74E2*, *uridine diphosphate glycosyltransferase 74E2*; *TAA1*, *L-trytophan-pyruvate. aminotransferase*; *AOP1*, *inactive 2-oxoglutarate-dependent dioxygenase AOP2*; *GAI*, *gibberellic acid insensitive*; *RGL*, *RGA* (*repressor of GA*)*-like*; *USP*, *universal stress protein*: *ABA2*, *ABA defcient 2*; *UGT71B6, UDP-glucosyl transferase 71B6*; *CKX3*, *cytokinin oxidase 3*; *UGT85A1*, *uridine diphosphate*

glycosyltransferase 85A1; *UGT*, *UDP-glucosyl transferase*; *IPT5*, *adenylate isopentenyltransferase 5*; *ARR*, *response regulator*; *AHP*: *histidine-containing phosphotransfer 4*; *UGT74F2*, *UDP-glucosyltransferase F2*; *ERF098*, *ethylene-responsive transcription factor 098*; *ACO1*, *ACC oxidase 1*; *SQE3*, *squalene epoxidase 3*; *CYP72A8*, *cytochrome P450*, *family 72*, *subfamily A*, *polypeptide 8*; *BGLU*, *β-glucosidase*; *EG*, *cellulose synthase-like*; *COBL*, *COBRA-like protein*; *CSLG*, *cellulose synthase-like G*; *XTH*, *xyloglucan endotransglucosylase*; *Xln*, *endo-1,4-beta-xylanase*; *Xyl*, *betaxylosidase*; *PL*, *pectate lyase*; *PE*, *pectinesterase;* *PG*, *polygalacturonase*; *PMEI*, *pectinesterase inhibitor*; *PNP*, *plant natriuretic peptide*; *EXP*, *expansin*; *AGP*, *arabinogalactan protein*; *ERG*, *glycine-rich cell wall structural protein*; *SKS*, *SKU5 similar 5*; *UGT79B6*, *UDP-glycosyltransferase 79B6-like*; *DFR*, *dihydrofavonol-4-reductase-like*; *DFRA*, *anthocyanidin reductase-like*; *UGT94E5*, *beta-D-glucosyl crocetin beta-1*, *6-glucosyltransferase-like*; *PKS5*, *polyketide synthase 5-like*; *BIS1*, *3,5-dihydroxybiphenyl synthase-like*; *AS*, *hydroquinone glucosyltransferase-like*; *CYP71A1*, *cytochrome P450 CYP736A12-like*; *SRG1*, *senescence-related gene 1*; *CODM*, *codeine O-demethylase-like*; *H6H*, *protein DOWNY MILDEW RESISTANCE 6-like*; *FLS*, *favonol synthase/favanone 3-hydroxylase-like*; *GT7*, *UDP-glucose favonoid 3-O-glucosyltransferase 7-like*; *F5H*, *ferulate 5-hydroxylase*; *CCoAOMT*, *Cafeoyl-CoA O-methytransferase*; *CCR*, *Cinnamoyl CoA reductase*; *CAD*, *cinnamyl alcohol dehydrogenase*; *POD*, *peroxidase*; *4CL*, *4 coumarate CoA ligase*; *HCT*, *hydroxycinnamoyl transferase*; *ABC*, *ATP Binding Cassette transporter*; *PIG*, *phosphatidylinositol-glycan biosynthesis protein*.

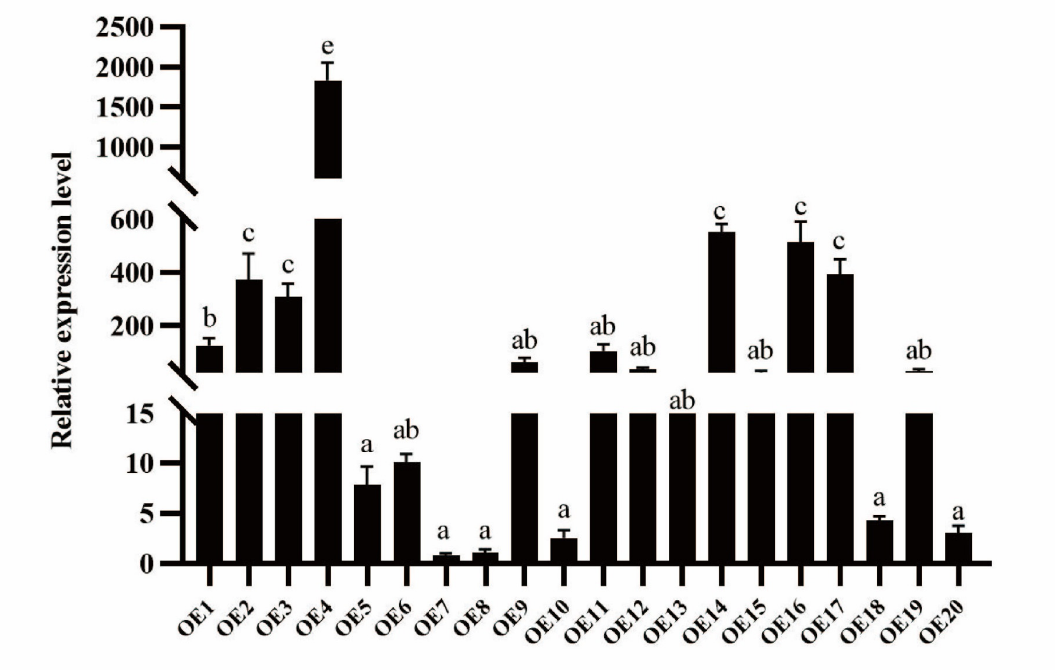

Fig S2. The results of identification of transgenic Arabidopsis positive seedlings. Expression levels of *PmGA2ox8* in the *Arabidopsis* lines.
